# Longer Colonoscopy Withdrawal Time Is Associated With the Detection of Visible Dysplasia in Patients With Inflammatory Bowel Disease

**DOI:** 10.1093/crocol/otae020

**Published:** 2024-03-23

**Authors:** Chandler McMillan, Darrick K Li, Gamal Mohamed, Danah A Alsadoun, Leena A Almohsen, Jill K J Gaidos, Deborah D Proctor, Badr Al-Bawardy

**Affiliations:** Yale School of Medicine, New Haven, CT, USA; Section of Digestive Diseases, Yale School of Medicine, New Haven, CT, USA; Department of Biostatistics, Epidemiology and Scientific Computing, King Faisal Specialist Hospital, Riyadh, Saudi Arabia; Department of Biostatistics, Epidemiology and Scientific Computing, King Faisal Specialist Hospital, Riyadh, Saudi Arabia; Department of Biostatistics, Epidemiology and Scientific Computing, King Faisal Specialist Hospital, Riyadh, Saudi Arabia; Section of Digestive Diseases, Yale School of Medicine, New Haven, CT, USA; Section of Digestive Diseases, Yale School of Medicine, New Haven, CT, USA; Section of Digestive Diseases, Yale School of Medicine, New Haven, CT, USA; Division of Gastroenterology and Hepatology, Department of Internal Medicine, King Faisal Specialist Hospital, Riyadh, Saudi Arabia; College of Medicine, Alfaisal University, Riyadh, Saudi Arabia

**Keywords:** inflammatory bowel disease, colonoscopy, dysplasia, polyp, withdrawal time

## Abstract

**Background:**

Colonoscopy withdrawal time (CWT) of at least 6–9 minutes is the minimum time needed for adequate adenoma detection in the general population. The ideal CWT in patients with inflammatory bowel disease (IBD) has not been determined. We aimed to identify the optimal CWT associated with the detection of visible dysplasia in patients with IBD.

**Methods:**

This is a retrospective study from 1/1/2017 to 9/1/2022 of adult patients with IBD in endoscopic healing undergoing surveillance via high-definition white light colonoscopy. The primary outcome was the association of CWT with visible dysplasia detection.

**Results:**

A total of 259 patients (mean age 56 ± 14.8 years; 51.3% female, 68% with ulcerative colitis; 8.9% with primary sclerosing cholangitis) underwent 330 colonoscopies. Patients with visible dysplasia were more likely to be older (*P* < .001) and have a personal history of visible dysplasia (*P* < .001) and invisible dysplasia (*P* = .023). The mean CWT was significantly longer in the visible dysplasia group at 26 minutes (interquartile range [IQR] 20–38.5) vs. 21 minutes (IQR 15–28) in procedures without visible dysplasia (*P* < .001). On multivariable analysis, increased age (*P* < .001), increased CWT (*P* = .001), and personal history of visible dysplasia (*P* = .013) were independently associated with the detection of visible dysplasia. A CWT of ≥15 minutes (odds ratio [OR] 2.71; 95% confidence interval [CI], 1.11–6.6; *P* = .02] and not ≥9 minutes (OR 2.57; 95% CI, 0.33–20.2; *P* = .35) is significantly associated with detection of visible dysplasia.

**Conclusions:**

For patients with IBD undergoing surveillance via high-definition white light colonoscopy, the mean CWT was independently associated with the detection of visible dysplasia.

## Introduction

Patients with inflammatory bowel disease (IBD) are at an increased risk of developing dysplasia and colorectal cancer (CRC) compared to the general population.^1,2^ CRC-related mortality is higher in patients with IBD and accounts for up to 10% of all deaths in patients with IBD.^3,4^ Colonoscopy is an important screening modality for the detection of dysplasia and thus routine surveillance colonoscopy is recommended in patients with ulcerative colitis (UC) and Crohn’s disease (CD) involving the colon starting 8–10 years after the initial diagnosis.^5,6^

In the general population, the majority of CRC develops from the adenoma to carcinoma progression pathway.^7^ Hence, the detection and removal of adenomas via polypectomy can reduce the incidence of CRC and decrease mortality by approximately 53% according to long-term follow-up studies.^8^ Longer colonoscopy withdrawal time (CWT), defined as the amount of time spent on inspection from cecal intubation to the time the colonoscope is withdrawn from the anal canal, is directly associated with increased adenoma detection.^9,10^

Multiple society guidelines have recommended a minimum CWT of 6 minutes during routine screening colonoscopies in the general population which has been deemed also as a quality indicator of the procedure.^11–13^ This was based on a landmark study by Barclay and colleagues in 2006 that demonstrated increased adenoma detection with a mean CWT greater than 6 minutes.^9^ More recently, several studies have demonstrated that a longer minimum CWT of 9 minutes is associated with improved rates of adenoma and polyp detection compared to 6 minutes.^14,15^

The association of CWT in patients with IBD undergoing surveillance colonoscopies and visible dysplasia detection has not been fully evaluated. In fact, many pivotal studies that investigated the significance of CWT in the general population excluded patients with IBD.^14,15^ Given the increased risk of CRC and CRC-related mortality in patients with IBD, prompting routine surveillance colonoscopy, further study of CWT and its significance is warranted in this population. Our study aims to evaluate the relationship between CWT and visible dysplasia detection in patients with IBD undergoing surveillance with high-definition white light colonoscopy.

## Materials and Methods

This was a single-center, retrospective review of all patients with IBD in endoscopic remission undergoing surveillance via high-definition white light colonoscopy from 6/1/2017 to 9/1/2022. The study protocol was approved by the Yale University Institutional Review Board (IRB#2000033392), and granted an official waiver of ethical approval.

We included patients at least 18 years of age who had a confirmed diagnosis of CD with colonic involvement, UC and IBD-unclassified involving the colon for ≥ 8 years, or a confirmed diagnosis of IBD and primary sclerosing cholangitis (PSC). We excluded patients with the following criteria: Active inflammation (defined as the presence of erosions and/or ulcers in CD, and a Mayo UC endoscopic subscore >1 in UC), and poor bowel preparation (defined as a Boston Bowel Preparation Scale <6 or bowel preparation categorized as “poor” by the performing endoscopist). We also excluded patients who underwent standard definition colonoscopy, dye chromoendoscopy, or patients with incomplete colonoscopy (defined as colonoscopies that failed to intubate the cecum) and patients with prior ileocolonic and/or colonic resections. Baseline characteristics and disease-related variables were extracted from patient charts including age, sex, race and ethnicity, body mass index, smoking status, personal history of invisible dysplasia and visible dysplasia, family history of CRC, IBD disease duration, subtype, location, phenotype, and current and previous IBD therapies.

Additional variables extracted from patient colonoscopy reports included credentials of the performing endoscopist (gastroenterology fellow, attending, IBD specialist), presence of pseudopolyps, presence of colonic strictures, number of polyps identified, and whether non-targeted biopsies were obtained. Pathology reports from each colonoscopy were reviewed to determine the presence of invisible and/or visible dysplasia. Invisible dysplasia was defined as the absence of an endoscopically visible lesion with pathology supporting indefinite, low-, or high-grade dysplasia. Visible dysplasia was defined as the presence of an endoscopically visible lesion (polypoid or non-polypoid) with pathology showing indefinite, low-, or high-grade dysplasia, adenoma, or sessile serrated lesion (SSL). CWT was defined as the time from cecal intubation to the withdrawal of the colonoscope from the anal canal, rounded to the nearest whole minute.

The primary outcome was to evaluate the association between CWT and the presence of visible dysplasia. The secondary outcome was to identify an optimal CWT cutoff associated with visible dysplasia detection.

All statistical analyses were performed using Stata version 17. For variables that were normally distributed, the data were summarized as mean and standard deviations. Variables that were not normally distributed were summarized as medians with interquartile ranges (IQRs). CWT cutoff values were tested for association with visible dysplasia detection. A CWT of 9 minutes was initially chosen as recent evidence has suggested that it is superior to a 6-minute withdrawal time in the general population.^14,15^ CWT was then divided into 3 groups using cutoff times of 9, 15, and 20 minutes. CWT, mean number of polypectomies, and patient characteristics were compared based on whether visible dysplasia was present or not. Unpaired *t*-tests were employed to compare continuous variables, whereas Pearson’s Chi-square test was used to compare categorical variables. Multivariable logistic regression analysis was performed to assess the joint association of patient characteristics with visible dysplasia. Results from logistic regression were presented as odds ratio (OR) with 95% confidence interval (CI). Receiver-operating characteristic curve (ROC) was used to determine the optimal cutoff point of CWT for detecting visible dysplasia detection. The area under the ROC curve (AUC) with its 95% CI was used to assess the predictive ability of CWT to detect visible dysplasia. Based on the cutoff point determined by the ROC curve and selected cutoff points for CWT (6, 9, 15, 20, and 25 minutes), the sensitivity and specificity of CWT to detect visible dysplasia were computed. A *P* value of <.05 was considered statistically significant.

### Ethical Considerations

This research study was conducted retrospectively from data obtained for clinical purposes. We consulted extensively with the Yale University Institutional Review Board who determined that our study did not need ethical approval. The Yale University Institutional Review Board granted an IRB official waiver of ethical approval.

## Results

A total of 259 patients underwent 330 colonoscopies and were included in the study. The mean age was 56 ± 14.8 years, and 51.3% of patients were female. Regarding IBD subtype, 30.9% of patients had CD, 68% had UC, and 1.1% had IBD unclassified. The median disease duration of IBD in the cohort was 15 years (IQR 8–24). A total of 23 (8.9%) patients had a diagnosis of PSC, and 15 (5.8%) patients were active smokers at the time of colonoscopy. A previous history of visible dysplasia was noted in 64 (24.7%), and 12 (4.6%) patients had a personal history of invisible dysplasia (Table 1). Inflammatory bowel disease specialists performed 135 (40.9%) colonoscopies, and 32 (9.7%) colonoscopies were performed by supervised gastroenterology fellows. Non-targeted biopsies were obtained in 321 (97.3%) colonoscopies performed. The median CWT for the cohort was 22 minutes (IQR 15–29).

**Table 1.** Baseline characteristics of patients with IBD included in the study.

Characteristic	Value
Age (years), mean (SD)	56.0 (14.8)
Female, *n* (%)	133 (51.3)
BMI, median (IQR)	27.4 (24–31.8)
Disease duration (years), median (IQR)	15 (8–24)
Ethnicity, *n* (%)	
Caucasian	205 (79.2)
Hispanic	18 (6.9)
African American	25 (9.7)
Asian	4 (1.5)
Others	6 (2.3)
Multiracial	1 (0.4)
IBD subtype	
Crohn’s disease, *n* (%)	80 (30.9)
L2	40
L3	40
B1	48
B2	13
B3	19
Ulcerative colitis, (%)	176 (68.0)
Proctitis	15
Left-sided colitis	63
Pancolitis	95
IBD-unclassified, *n* (%)	3 (1.1)
Primary sclerosing cholangitis, *n* (%)	23 (8.9)
Personal history of visible dysplasia, *n* (%)	64 (24.7)
Personal history of invisible dysplasia, *n* (%)	12 (4.6)
Family history of colorectal cancer, *n* (%)	26 (10.0)
Smoking, *n* (%)	15 (5.8)
Oral mesalamine, *n* (%)	125 (48.3)
On biologic, *n* (%)	69 (26.7)
Infliximab, *n*	34
Adalimumab, *n*	14
Vedolizumab, *n*	14
Ustekinumab, n	6
Certolizumab pegol, n	1
Tofacitinib, *n* (%)	4 (1.5)
On immunomodulator	47 (18.1)
Thiopurine	42
Methotrexate	5

Abbreviations: IBD, inflammatory bowel disease; IQR, interquartile range; SD, standard deviation.

**Figure 1.**
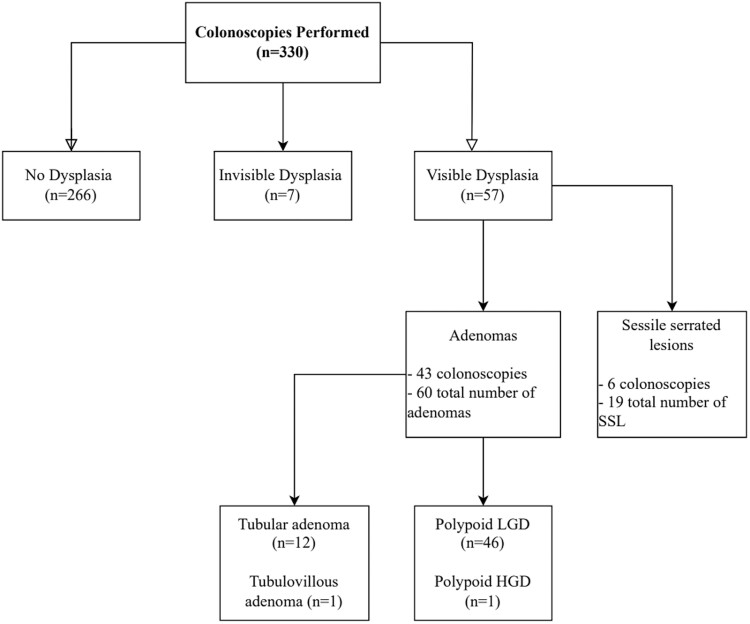
Flowchart diagram illustrating the findings of the colonoscopies performed and type of dysplasia identified. Two colonoscopies had both adenoma and sessile serrated lesion (SSL). LGD, low-grade dysplasia; HGD, high-grade dysplasia.

Invisible dysplasia was detected in 2.1% (*n* = 7). Of these 7 colonoscopies, 1 had visible dysplasia also. The overall rate of visible dysplasia detection in the sample was 17.3% (*n* = 57). This represents the proportion of colonoscopies that detected at least 1 type of visible dysplasia. Polypectomies were performed in all 57 colonoscopies with visible dysplasia detection. Adenomas were detected in 43 of the procedures while SSLs were detected in 16 procedures (2 procedures had both adenoma and SSLs detected). A total of 60 adenomas and 19 SSLs were detected in these colonoscopies. Of the 60 adenomas, 1 was noted to be polyploid high-grade dysplasia, 1 was tubulovillous adenoma, 12 were tubular adenomas, and 46 were polypoid low-grade dysplasia (Figure 1). Differences in baseline and disease characteristics between the groups with and without visible dysplasia detection are shown in Table 2. Compared to patients without visible dysplasia, patients with visible dysplasia were older (*P* < .001) and more likely to have a personal history of visible dysplasia (*P* < .001) and invisible dysplasia (*P* = .023). There were no significant differences between the 2 groups when we assessed procedural factors, such as bowel preparation, presence of pseudopolyps and colonic stricture, as well as proceduralist factors including the presence of an IBD specialist or gastroenterology fellow (Table 3).

**Table 2.** Comparison of characteristics between patients with and without visible dysplasia.

	Visible dysplasia (*n* = 45 patients)	No visible dysplasia (*n* = 214 patients)	*P* value
Baseline characteristics			
Age (years), mean (SD)	62.9 (12.5)	54.6 (14.9)	**<.001**
Female sex, *n* (%)	20 (44.4)	113 (52.8)	.31
Male sex, *n* (%)	25 (55.6)	101(47.2)	
Smoking, *n* (%)	3 (6.7)	11 (5.1)	.68
Family history of CRC, *n* (%)	4 (8.9)	23 (10.8)	.71
Disease duration (years), median (IQR)	14 (8-24)	15 (8-24)	.71
Disease characteristics			
Disease type, *n* (%)			
Crohn’s disease	12 (26.7)	68 (31.8)	
Ulcerative colitis	33 (73.3)	143 (66.8)	.56
Indeterminate colitis	0 (0)	3 (1.4)	
Primary sclerosing cholangitis, *n* (%)	2 (4.4)	20 (9.4)	.28
Personal history of visible dysplasia, *n* (%)	21(46.7)	43(20.1)	**<.001**
Personal history of invisible dysplasia, *n* (%)	5 (11.1)	7 (3.3)	**.02**
Medications			
On mesalamine at time of colonoscopy, *n* (%)	19 (42.2)	103 (48.1)	.47
On biologic at time of colonoscopy, *n* (%)	10 (22.2)	62 (28.9)	.36
Steroids at time of colonoscopy, *n* (%)	3 (6.7)	11 (5.1)	.68
On tofacitinib, *n* (%)	1 (2.2)	3 (1.4)	.69
On immunomodulator, *n* (%)	5 (11.1)	40 (18.7)	.22

Abbreviations: CRC, colorectal cancer; IBD, inflammatory bowel disease; IQR, interquartile range; SD, standard deviation.

Bold indicates *P* value <.05.

**Table 3.** Comparison of procedural factors among colonoscopies with and without visible dysplasia detection.

	Visible dysplasia (*n* = 57 colonoscopies)	No visible dysplasia (*n* = 273 colonoscopies)	*P* value
Procedure factors			
Excellent/good bowel preparation, *n* (%)	49 (85.9)	251 (91.9)	.15
Withdrawal time, median (IQR)	26 (20-38)	21 (15-28)	**<.001**
Pseudopolyps, *n* (%)	10 (17.5)	41 (15.0)	.63
Colonic stricture, *n* (%)	1 (1.8)	5 (1.8)	.97
Proceduralist factors			
IBD specialist, *n* (%)	20 (35.1)	115 (42.1)	.33
GI fellow, *n* (%)	7 (12.3)	25 (9.2)	.47

Abbreviations: GI, gastrointestinal; IBD, inflammatory bowel disease; GI, gastroenterology.

Bold indicates *P* value <.05.

A total of 23 patients (8.9%) had a diagnosis of PSC and underwent 34 colonoscopies in the study. Non-targeted biopsies were obtained in 94.1% (*n* = 32) of colonoscopies performed in PSC patients. Invisible dysplasia was detected in 2.9% (1/34) of colonoscopies performed in PSC patients compared to 2.0% (6/296) in colonoscopies of non-PSC patients.

**Figure 2.**
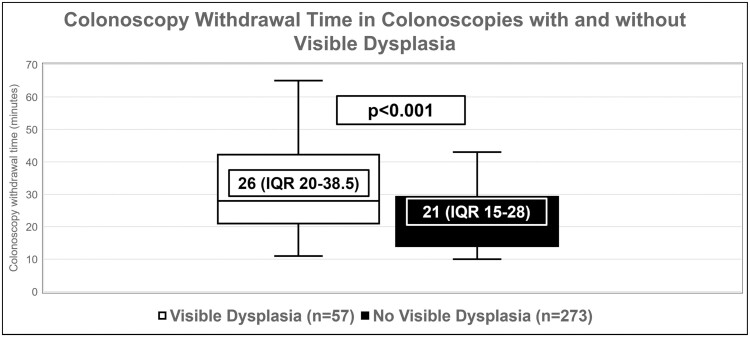
Box plot comparing colonoscopy withdrawal time in the group with and without visible dysplasia.

The CWT was significantly higher in the visible dysplasia group at 26 minutes (IQR 20–38.5) compared to a median CWT of 21 minutes (IQR 15–28) for colonoscopies that did not detect visible dysplasia (*P* < .001) (Figure 2). At least 1 polypectomy was performed in 74 colonoscopies in the group without visible dysplasia where pathology was consistent with hyperplastic polyps. The mean number of polypectomies was 2.1 ± 1.5 in this group of colonoscopies (*n* = 74) and 2.3 ± 2.3 in the group with visible dysplasia (*n* = 57) (*P* = 0.71). The median withdrawal time in colonoscopies in which SSLs were detected was 25 minutes (IQR 21.3–48.8), while the median withdrawal time in colonoscopies in which adenomas were detected was 27 minutes (IQR 20–38).

**Figure 3.**
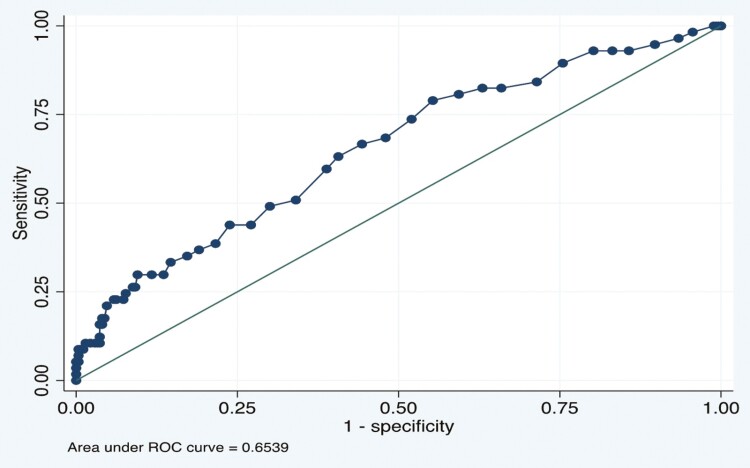
Receiver-operating characteristics curve of withdrawal time and visible dysplasia detection in inflammatory bowel disease.

On multivariable analysis, increasing age (OR 1.04; 95% CI, 1.02–1.07, *P* < .001), longer CWT (OR 1.04; 95% CI, 1.02–1.06, *P* = .001), and personal history of visible dysplasia (OR 2.24; 95% CI, 1.18–4.25, *P* = .013) were all independently associated with an increased odds of visible dysplasia detection (Table 4). Colonoscopy withdrawal time cutoffs of ≥15 minutes (OR 2.71; 95% CI, 1.11–6.60, *P* = .02) and ≥20 minutes (OR 3.02; 95% CI, 1.53–5.98, *P* < .001) were significantly associated with the detection of visible dysplasia. Conversely, a CWT cutoff of ≥9 minutes (OR 2.57; 95% CI, 0.33–20.2, *P* = .35) was not significantly associated with the detection of visible dysplasia. Assessing CWT as a continuous variable, we found that for every 1-minute increase in CWT, there was a subsequent 4.2% increase in visible dysplasia detection (OR 1.04; 95% CI, 1.02–1.06; *P* = .001). An ROC curve demonstrated an AUC of 0.65 (95% CI, 0.57–0.73) for a CWT cutoff of 23 minutes (Figure 3).

**Table 4.** Multivariable regression analysis of factors associated with visible dysplasia.

Variables	Odds ratios	95% CI	*P* value
Age (years)	1.04	1.02–1.07	**<.001**
Disease duration (years)	0.98	0.96–1.00	.11
Withdrawal time (minutes)	1.04	1.02–1.06	**.001**
Personal history of visible dysplasia	2.24	1.18–4.25	**.013**
Personal history of invisible dysplasia	1.47	0.47–4.62	.163

Abbreviations: 95% CI, 95% confidence interval.

Bold indicates *P* value <.05.

## Discussion

In this study, we evaluated the relationship between CWT and the rate of visible dysplasia detection in patients with IBD. The overall rate of visible dysplasia detection in our cohort was 17.3%, and multivariable analyses demonstrated that longer CWT is independently associated with visible dysplasia detection in patients with IBD. Specifically, a CWT cutoff ≥15 minutes was significantly associated with visible dysplasia detection in this group of patients with IBD. Conversely, a CWT cutoff of ≥9 minutes was not significantly associated with visible dysplasia detection.

In the general population, multiple society guidelines and consensus statements have recommended a minimum CWT of at least 6 minutes.^11–13^ The landmark study conducted by Barclay et al. in 2006 evaluated CWT and adenoma detection rate for 12 endoscopists and determined that endoscopists with a mean CWT ≥6 minutes had significantly higher rates of adenoma, advanced adenoma, and neoplasia detection compared to endoscopists with a mean CWT < 6 minutes.^9^ More recent prospective studies in the general population have proposed a longer CWT cutoff of 9 minutes for optimal detection of adenomas and polyps.^14–16^ These studies have excluded patients with IBD and hence it is unclear if this ideal CWT cutoff time is applicable to patients with IBD.

Recent studies have demonstrated a reduction in the rate of CRC incidence in IBD, likely owing to improvement in medical management and surveillance.^17,18^ However, the relative risk of developing CRC in UC and colonic CD is 2-fold compared to the general population.^3^ In addition, the burden of CRC in IBD is not trivial as it as it responsible for 10%–15% of the annual mortality in patients with IBD.^5^ Patients with IBD are also prone to developing dysplasia in both endoscopically visible and invisible lesions, and the presence of low-grade dysplasia in this population confers a 9-fold increase in risk of neoplasia development compared to a 1.8-fold risk of neoplasia in the general population.^19,20^ Given the increased risk for transformation of low-grade dysplasia to neoplasia in patients with IBD, surveillance colonoscopy requires meticulous inspection of the mucosa, identification, and removal of dysplastic lesions when possible. Thus, patients with IBD may require longer average CWTs compared to patients undergoing screening colonoscopy without history of colonic inflammation.

In our cohort, the rates of invisible and visible dysplasia were 2.1% and 17.3%. This is similar to previous reports including a population-based study that showed a total prevalence of neoplasia associated with UC to be at 23.6% (this included adenomas and CRC).^21^ Risk factors for colonic dysplasia associated with IBD include disease duration, extent, cumulative inflammation, smoking, age, family history, and PSC.^22,23^ The patient population included in this study was relatively at higher risk for colonic neoplasia with a mean age of 56 years, median disease duration of 15 years, and almost 9% with concomitant PSC. We did not have any cases of colon cancer in this cohort and that is likely due to patient selection per predetermined criteria. We did not include all consecutive patients with IBD undergoing surveillance colonoscopy as we excluded patients who had active inflammation or suboptimal bowel preparation.

Our study focuses on CWT and visible dysplasia detection in patients with IBD specifically as this population is at higher risk of CRC compared to the general population. There are no previous studies looking at the relationship between CWT and dysplasia detection in IBD. A recent systematic review of quality metrics in IBD surveillance colonoscopies suggested at least a 17-minute withdrawal time when performing dye chromoendoscopy.^24^ This was based on an average of an additional 11 minutes needed during withdrawal of dye chromoendoscopy exams which would be dedicated to washing the mucosa, applying the dye, careful inspection of the mucosa, and suction of fluid.^25^ However, no clear consensus or recommendation was made as to the ideal minimum withdrawal time for high-definition white light colonoscopy surveillance exams in patients with IBD. Therefore, our findings offer new insight regarding this important quality indicator and predictor of adenoma detection. We have found that a minimum withdrawal time of at least 15 minutes and not 9 minutes is associated with visible dysplasia detection. We were also able to demonstrate that CWT is an independent predictor of visible dysplasia detection.

There are multiple strengths to our study. We have included a homogenous population of patients by selecting those in endoscopic remission and adequate bowel preparation to limit confounders of CWT. We also have a relatively high number of colonoscopies included in the study (>300). Our study is the first to examine the relationship of CWT during high-definition white light colonoscopy surveillance exams in patients with IBD. Our study also has several limitations. For one, we are not able to account for the time taken to perform biopsies or polypectomies. However, we did note that there were 74 colonoscopies in the non-dysplasia group in which polypectomies were performed compared to the 57 in the dysplasia group with a similar median number of polypectomies of 2 per each colonoscopy in both groups. In addition, it is possible that clinicians may perform a more thorough withdrawal examination on colonoscopy when cognizant of a patient’s prior history of visible and/or invisible dysplasia, which could potentially contribute to a higher dysplasia detection rate and longer withdrawal times. We also could not account for the utilization of narrow-band imaging or virtual chromoendoscopy in the procedures included in the study. Lastly, our cohort reflects that of a tertiary referral center who were at a relatively higher risk of dysplasia and hence results might not be generalizable.

## Conclusions

In conclusion, our study found that CWT was independently associated with visible dysplasia detection in IBD surveillance exams. Specifically, a CWT cutoff of ≥15 minutes was significantly associated with visible dysplasia detection, while a CWT of ≥9 minutes was not. Every 1-minute increase in withdrawal time was associated with a 4.2% increase in visible dysplasia detection. Future directions include prospective controlled studies to better ascertain the relationship between CWT and visible dysplasia detection in patients with IBD.

## Data Availability

Data not publicly available.
